# Nontechnical skills training in intensive care units: Protocol for a systematic review and meta-analysis

**DOI:** 10.1371/journal.pone.0280132

**Published:** 2023-01-06

**Authors:** Isac Davidson Santiago Fernandes Pimenta, Ádala Nayana de Sousa Mata, Isaac Newton Machado Bezerra, Helaine Carneiro Capucho, Adriana Catarina de Souza Oliveira, Paloma Echevarría Pérez, Grasiela Piuvezam

**Affiliations:** 1 Post-Graduation Program in Public Health, Federal University of Rio Grande do Norte, Natal, Brazil; 2 Multicampi School of Medical Sciences of Rio Grande do Norte, Federal University of Rio Grande do Norte, Caicó, Brazil; 3 Vitoria Academic Center, Federal University of Pernambuco, Vitoria do Santo Antão, Brazil; 4 Department of Pharmacy, Faculty of Health Sciences, University of Brasilia, Brasilia, Brazil; 5 Faculty of Nursing, Catholic University of Murcia, Murcia, Spain; 6 Department of Public Health, Federal University of Rio Grande do Norte, Natal, Brazil; Xiamen University - Malaysia Campus: Xiamen University - Malaysia, MALAYSIA

## Abstract

This study is aimed at describing a protocol for a systematic review and meta-analysis to assess the effect of nontechnical skills training on the acquisition of knowledge, skills or attitudes, and changes in behavior at the workplace, of healthcare professionals working in intensive care units (ICUs), as well as the effect on outcomes at an organizational level. We will search for original studies in the PubMed/Medline, Scopus, Web of Science, Science Direct, EMBASE and PsycINFO databases. Studies with a clinical trial or quasi-experimental design will be included. Two reviewers will independently screen and assess the included studies, with any disagreements being resolved by a third reviewer. We will summarize the findings using a narrative approach and, if possible, conduct a quantitative synthesis (meta-analysis). We will conduct the protocol following the Preferred Reporting Items for Systematic Review and Meta-Analyses Protocols (PRISMA-P) guidelines. The review will summarize the current evidence on nontechnical skills training in ICUs, examining satisfaction with the training program, improvements in knowledge about nontechnical skills and the adoption of safety behaviors, as well as improvement in outcomes for the organization, such as mortality rates, length of stay and cost indicators. We expect that the systematic review could indicate effective strategies for training ICU professionals in nontechnical skills and also determine whether these strategies really improve the safety culture and professional knowledge and behaviors, as well as patient outcomes and safety.

## Introduction

Nontechnical skills (NTS), defined as ‘the cognitive, social and personal resource skills that complement technical skills, and contribute to safe and efficient task performance’ [1], have an important role in many high-reliability organizations, such as the aviation, nuclear and oil industries [1, 2].

Similar to these organizations, healthcare services have a complex socio-technical environment with a higher risk of errors [2]. Estimates indicate that at least 9% of hospital admissions are complicated by safety incidents, with a large number of them being preventable [3]. In intensive care units (ICUs), the prevalence of such events is higher than in other medical settings [4].

Analysis of incident reports in ICUs shows that human factors are an important underlying condition in safety incidents [5]. NTS such as teamwork, leadership, situational awareness, decision-making and task management are some of these behaviors [1, 5]. In a literature review by Reader et al. [6] at least 50% of the contributory factors in safety incidents were NTS related.

In light of this, NTS training seems to be especially valuable in ICU environments, it being necessary to identify the effect of training programs on health professionals’ performance. To evaluate the effects of training interventions in a work context, Kirkpatrick [7] propose a scale with four levels: *reaction*–which refers to the participant’s feedback of training experience; *learning*–the acquisition of knowledge, skills or attitudes by participants after training; *behavior*–changes in participant behavior at the workplace; and *results*–the measurable impact of training at an organizational level.

Some studies indicate that the implementation of NTS training in ICUs may provide better performance to the professionals and reduction of clinical complications to the patients. Mayer et al. [8] implemented TeamSTEPPS^®^ training in two ICUs in a before-and-after study, and found significantly higher scores for observed behaviors such as team performance, leadership and mutual support after the intervention. At an organizational level, the average time for placing patients on extracorporeal membrane oxygenation (ECMO) was reduced, despite having a small effect size.

Haerkens et al. [9] implemented crew resource management training during a 3-year prospective study in an ICU with 2500–3000 admissions per year. The found an association between the implementation of the training program and a significant reduction of overall complication rate, from 66.4 at baseline to 50.9/1000 patients in the post-implementation year. In the same period, the cardiopulmonary resuscitation success rate increased from 19% to 67%, also associate with the intervention. However, none of the studies used a randomized and controlled design, which may affect the magnitude of the results.

Nevertheless, the impact of poor performance of NTS skills presents in safety incidents in healthcare services are very recognizable [6, 10] and no systematic review has investigated the effect of NTS training programs in the ICU context. Thus, this paper describes a protocol for a systematic review and meta-analysis that aims to assess the effect of NTS training on the reaction, knowledge and behavior of healthcare professionals working in ICUs, as well as its effect on outcomes at an organizational level.

## Methods and analysis

### Study registration and reporting

This systematic review study is registered on the PROSPERO database under number CRD42021244769. The protocol is based on the Preferred Reporting Items for Systematic Reviews and Meta-Analyses Protocols (PRISMA-P) guidelines [11]. The final report will be developed following PRISMA [12] and the *Cochrane Handbook for Systematic Reviews of Interventions* [13] and any changes to the protocol will be described in the Method section.

### Eligibility criteria

#### Types of studies

We will include randomized and nonrandomized clinical trials, quasi-experimental studies with a control group, interrupted time series studies and before-and-after studies without a control group.

#### Types of participants

We will include studies with healthcare professionals working in ICUs, such as physicians, nurses, pharmacists, and respiratory therapists. We will also consider studies that include residents and interns, but only if they are integrated into the ICU staff.

#### Intervention

We will consider all types of training programs that aim to improve NTS performance in a workspace context if they report data both before and after the intervention. For the purpose of this review, NTS are cognitive, social and personal skills that complement technical skills such as teamwork, situational awareness, task management and decision-making.

We will only consider interventions that focus on training a set of NTS, considering that being proficient in only a single skill is insufficient to improve safety in high-risk environments such as ICUs [1, 6].

The intervention could be delivered by a single methodology or by a combination of methods, such as e-learning modules, simulations, high-fidelity simulations, lectures or workshops. Furthermore, we will consider interventions of any duration and frequency.

#### Comparison

We will consider studies with a control group that compare the intervention with no training or training in a different set of skills.

#### Outcomes

The outcome measures will consider the four levels of Kirkpatrick’s scale [7]: *reaction*–participant’s feedback of training experience; *learning*–the acquisition of knowledge, skills or attitudes by participants after training; *behavior*–changes in participant behavior at the workplace; and *results*–the measurable impact of training at an organizational level.

As there are a variety of measures for each level of Kirkpatrick’s scale, the included studies may consider the following outcomes: indicators of satisfaction with the training program; mean scores in training assessments; changes in safety behaviors (e.g. teamwork, situational awareness); prevalence of adverse events or patient safety incidents; adoption of good practices or standard practices; morbidity or mortality rates; and cost indicators (e.g. return of investment rates).

#### Exclusion criteria

We will exclude studies that: were implemented only with managers, administrators or leaders; provide an incomplete description of the training intervention or evaluation method; involved a large improvement strategy with multiple interventions; used coaching or mentoring intervention strategies; were pilot, validation or qualitative studies. Theses, reviews, cases reports, conference papers and abstracts were also excluded.

### Search strategy

The systematic review will summarize the evidence of primary studies in the following databases: PubMed/Medline; Scopus; Web of Science; Science Direct; EMBASE, CINHAL and PsycINFO. In addition, the references of the included studies will be screened to search for other relevant studies that do not appear in the main search. No restrictions in terms of language or year of publication will be made. We will not search in gray literature.

The search strategy combined terms from the Medical Subject Headings (MeSH) and Embase Subject Headings (EMTREE), as well as using non-MeSH terms. Considering the absence of a specific framework for NTS in ICUs, the non-MeSH terms are selected based on the Anesthetists’ Non-Technical Skills (ANTS) system [14]. This framework is largely used across acute medical settings beyond anesthesia and is consistent enough to be used in ICUs [6]. Also, the NTS needed in various high-risk domains seem to be very analogous, considering that human behavior across these settings is also very similar [1].

The primary search strategy resulted in the following combination of keywords: “Non-technical skill*” OR "decision mak*" OR "Leader*" OR "Team Work*" OR "Situational Awareness" OR "Human factor*" AND "Intensive Care Unit*" AND "Training".

### Study selection

After extraction of the records in all the databases, we will make an initial check for duplicates, following their proper removal. Two reviewers will independently screen the records by reading the titles and abstracts, and then read the full text to assess the eligibility criteria. Any disagreements between reviewers in any phase of study selection will be resolved by consulting a third reviewer.

To perform all phases of the study selection, we will use the Rayyan^®^ application for systematic reviews [15].

### Data extraction and management

Two reviewers will extract the data independently, using a standard electronic spreadsheet previously tested. The data will include identification of the studies (aim, year, main author, country, ICU type and study design), population characteristics (age, gender, profession), description of the intervention (educational strategy, duration and curricula), method of educational assessment (questionnaires, method of observation) and outcomes (mortality rates, cost indicators, length of stay, incidence of safety incidents, indicators of satisfaction with the training program, improvement in knowledge or safety behavior).

### Dealing with missing data

In the case of interesting data missing or being unclear, the research team will try to contact the corresponding author by email, phone or correspondence. If this communication is unsuccessful, we will exclude the data from the analysis, covering this in the Discussion section.

### Risk of bias and quality assessment

Two reviewers will independently assess the selected studies, using Version 2 of the Cochrane Risk of Bias (ROB 2) tool for randomized clinical trials or the Risk of Bias in Non-randomized Studies of Interventions (ROBINS-I) for nonrandomized clinical trials and quasi-experimental studies with a control group, classifying the risk of bias as low, high or unclear [13]. The EPOC Risk of Bias tool will be used to assess bias in interrupted time series studies and the National Heart, Lung, and Blood Institute quality assessment tool will be used for before-and-after studies without a control group [16].

In addition, the Grading of Recommendations Assessment, Development and Evaluation (GRADE) tool will be used to assess the quality of evidence [17]. To calculate the inter-rater reliability, we will use the kappa coefficient.

### Data synthesis

We will carry out a narrative synthesis and produce summarizing tables considering the data extraction plan. Dichotomous outcomes will be summarized as risk ratios with 95% confidence interval (CI), whereas continuous outcomes will be summarized as mean differences with 95%CI. The *p* value of each result will also be considered [18].

If the included studies are methodologically homogeneous, we will conduct a meta-analysis. To verify the heterogeneity between studies, we will use the χ^2^ test with a significance of *p* < 0.05. In addition, the I^2^ statistic will be calculated to assess the consistency between studies, considering a value of 0% as no observed heterogeneity, up to 50% as a moderate level and 75% or higher as a substantial level of heterogeneity. If possible, we will perform a funnel plot analysis to indicate possible reporting biases and a linear regression approach to measure the asymmetry of the funnel plot. Review Manager software (RevMan V5.3.3) will be used for the data analysis.

### Dissemination and ethics

We will publish the results of the systematic review in a peer-reviewed journal. In addition, the results will be disseminated in academic and health service spaces, such as at conferences or seminars. No ethical committee approval is required, given that the review will not use the personal data of professionals or patients.

## Discussion

This protocol proposes a systematic review to summarize the current evidence on the effect of NTS training programs in ICUs from a professional perspective, as well for the patients and healthcare organizations.

Despite the technological and scientific advances in clinical practices, delivering high-quality healthcare services is still a major challenge to governments and organizations, especially with regard to patient safety [19, 20].

Inspired by high-reliability organizations, healthcare services incorporate many techniques and approaches to understand and solve safety problems. Understanding the role of human factors in patient safety is one of these approaches [21]. The literature demonstrates a close relation between poor NTS and safety incidents in ICUs [5, 6, 10]. However, reports about NTS training programs have particularly focused on surgical specialties [22–24].

### Implications

The NTS training can help health professionals to perform procedures more safely and efficiently and are useful in daily clinical practice in ICUs [1, 6, 8, 9]. Skills such as gathering and interpreting information, anticipating future status, defining problems and selecting courses of action, and reviewing outcomes, are essential for every care process and should be addressed by training programs [1, 6, 14].

Safety incidents in ICUs are often related to processes such as medication preparations and administration, and the use of equipment and tubes [25, 26]. Incorporing or enhance NTS in clinical practice may help healthcare professionals to conduct procedures more safely, reducing avoidable safety incidents and providing better outcomes for the patients, such as reducing the length of stay and care-related infections, for example [8, 9].

In light of this, systematic reviews play an important role in summing up multiple initiatives for quality improvement and highlight the better practices to adopt when considering the variety of contexts [19, 27, 28]. We expect that this review could help healthcare services to implement NTS training in the ICU context, supporting the choices with evidence for more effective teaching methods that are well received by the professionals.

### Limitations

We may find some limitations with this review. The inclusion of studies with before-and-after designs can compromise the quality of evidence provided by the review. This variety of study types included can also generate heterogeneity between studies, which could compromise a quantitative summary of the results (meta-analysis).

However, in quality improvement initiatives, as in Plan-Do-Study-Act cycles for example, randomized and controlled designs may not be an option due to limitations such as resource availability and ethical questions [29, 30]. In such context, nonrandomized studies assume a very important role to overcome this limitations and understand the effect of an intervention in clinical outcomes [31].

Futhermore, some methods of evaluation for each outcome could also vary, considering the large availability of instruments for measuring NTS in very different ways, such self-reported questionaries, and direct observation, for example [14].

Despite of this limitations, this research will provide an important overview of the use of NTS training programs in the ICUs context, that can colaborate with further interventions to improve care quality and safety.

## Supporting information

S1 Checklist PRISMA-P (Preferred Reporting Items for Systematic review and Meta-Analysis Protocols) 2015 checklist: Recommended items to address in a systematic review protocol. (DOC)
